# In vivo kinematics of a unique posterior-stabilized knee implant during a stepping exercise

**DOI:** 10.1186/s13018-016-0354-5

**Published:** 2016-02-01

**Authors:** Takatomo Mine, Kenji Hoshi, Kazuyoshi Gamada, Koichiro Ihara, Hiroyuki Kawamura, Ryutaro Kuriyama, Ryo Date

**Affiliations:** Department of Orthopaedic Surgery, National Hospital Organization Kanmon Medical Center, 1-1 ChofuUshiroda Simonoseki, Yamaguchi, 752-8510 Japan; Department of Rehabilitation, Hiroshima International University, Hiroshima, Japan

**Keywords:** Kinematics, Stair-stepping motion, Total knee arthroplasty, Unique posterior-stabilized knee implant

## Abstract

**Background:**

Stair-stepping motion is important in daily living, similar to gait. Knee prostheses need to have even more superior performance and stability in stair-stepping motion than in gait. The purpose of this analysis was to estimate in vivo knee motion in stair stepping and determine if this unique knee prosthesis function as designed.

**Methods:**

A total of 20 patients with Bi-Surface posterior-stabilizing (PS) implants were assessed. The Bi-Surface PS knee is a posterior-cruciate substitute prosthesis with a unique ball-and-socket joint in the mid-posterior portion of the femoral and tibial components. Patients were examined during stair-stepping motion using a 2-dimensional to 3-dimensional registration technique.

**Results:**

The kinematic pattern in step up was a medial pivot, in which the level of anteroposterior translation was very small. In step down, the kinematic pattern was neither a pivot shift nor a rollback. From minimum to maximum flexion, anterior femoral translation occurred slightly.

**Conclusions:**

In this study, this unique implant had good joint stability during stair stepping. The joint’s stability during stair stepping was affected by the design of the femorotibial joint rather than post/cam engagement or the ball-and-socket joint.

## Background

**Fig. 1:**
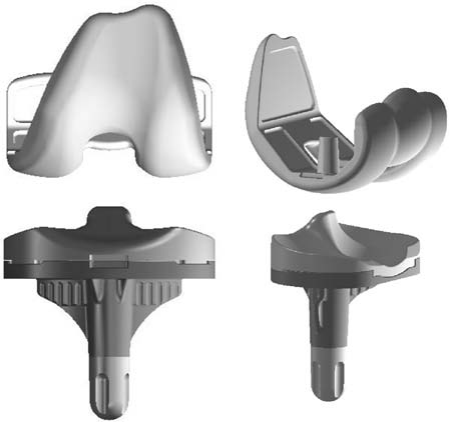
Bi-surface PS type

Clinical and radiographic examinations are commonly used to evaluate the postoperative outcomes of total knee arthroplasty (TKA). During such assessments, kinetic and gait analyses are considered to be essential for determining the detailed effects of TKA. In particular, fluoroscopic in vivo kinematic studies performed during knee flexion have been demonstrated to be useful for assessing the postoperative outcomes of TKA [1–5]. Knee motion patterns have been examined in various studies of gait, step, stair, or deep bending-based activities. During daily activities, knee implants partially replicate the intrinsic constraints of the original joint. Many different types of knee implants have been developed. The Bi-Surface posterior-stabilizing (PS) knee implant (Kyocera) was designed to improve the range of deep flexion and stability, and its mid-posterior portion contains a ball-and-socket joint that links its femoral and tibial components. This characteristic structure allows a larger contact area between the femoral and tibial articular surfaces and reduces the stress placed on the tibial plate. In addition, the articular surface of the tibial plate is asymmetric; it is concave on the medial side and flat on the lateral side. The post/cam mechanism of the Bi-Surface PS implant is designed to enable it to function from 45° to 60° of knee flexion during stair stepping, which allows the femoral component to roll back early (Fig. 1).

Among daily activities, the ability to use the stairs is very important, as is gait. It is more important for knee prostheses to exhibit good performance and stability during stair stepping than during walking. Therefore, it is important to understand the relationship between implant design and functional knee motion during stair stepping. The goal of this analysis was to assess in vivo knee motion during a stepping exercise and determine the motion pattern in patients with Bi-Surface PS knee implant functions.

The 3-dimensional (3D) positioning and orientation of the implant components were determined using a 2D/3D registration technique involving previously reported methods, manual matching, and image space optimization [1–3, 6]. Using this approach, we performed an in vivo kinematic analysis of stepping activity in patients that had been implanted with the Bi-Surface PS knee prosthesis.

## Methods

Twenty subjects that underwent TKA involving a Kyocera Bi-Surface PS knee prosthesis (Kyocera, Japan) were assessed in this study. The patients had undergone clinically successful TKA and were willing to participate in this study. The patients were followed up for more than 6 months before being assessed and included 18 females and 2 males. All of the patients had been diagnosed with osteoarthritis. Their mean age was 74.7 years (range 64–83). All of the TKA procedures were performed by the same surgeon, and a parapatellar approach was used in all cases. The patella was not resurfaced, and all of the implants were fixed in place with cement. At the time of the analysis, the mean duration of the postoperative follow-up period was 7.1 ± 1.2 months (range 6–11). Clinical evaluations were performed according to the knee rating scale of the Hospital for Special Surgery (HSS) after arthroplasty. The mean postoperative HSS score was 91.9 ± 3.3 (range 86–97). This study was conducted in accordance with the Declaration of Helsinki and with approval from the Ethics Committee of Kanmon Medical Center (Shimonoseki, Japan). Written informed consent was obtained from all participants or their guardians.

Each patient was examined under fluoroscopic surveillance in the sagittal plane whilst stepping onto and off a 10-cm-high step. During the examinations, the patients stood with their feet in neutral rotation. Then, they stepped onto and off the step. Both of these movements were performed using a single leg. The patients began by placing their ipsilateral foot onto the 10-cm-high step. They were then instructed to step up onto the step, before swinging their other leg through and onto the step. When stepping down, they were instructed to step off the step with their opposite leg and stand with the ipsilateral foot remaining on the 10-cm-high step. Three successful sets of movements were recorded, and the best recording was used for the analysis. Successive knee motions were recorded as serial digital x-ray images (2048 × 1536 × 14 bits/pixel, 194-μm serial spot images, saved as DICOM files) using a 40 cm × 30 cm flat panel detector system (DHF-155H3, Hitachi, Japan) and 1.2- to 2.0-ms pulsed x-ray beams. The 3D in vivo positions of the Bi-Surface prosthesis were computed at 10° intervals using a 2D/3D registration technique. The digital fluoroscopic images were undistorted using a custom MATLAB program. The optical geometry of the fluoroscopic system (principal distance, principal point) was determined based on images of a calibration target [3, 4]. An implant surface model was projected onto the geometry-corrected fluoroscopic images, and its 3D position was iteratively adjusted so that its silhouette matched with that of the knee prosthesis using custom software (JointTrack, University of Florida, FL). After the matching procedure had been completed, videos of the movements of the bone model and the 6 degrees of freedom kinematics of the implant components were acquired and subjected to quantitative analysis (3D-JointManager, GLAB Inc., Hiroshima, Japan). The matching procedure exhibited standard errors of approximately 0.5° to 1.0° for rotations and 0.5 to 1.0 mm for translations in the sagittal plane [4]. The relative movements of the femoral and tibial components were determined based on the 3D positions of the knee prosthesis using the projection coordinate system proposed by Andriacchi [7].

We evaluated the flexion angle, the axial rotation angle, anteroposterior translation, the valgus/varus angle, and post/cam engagement between the femoral and tibial components during stepping up and down movements. In patients with fixed-bearing knee prostheses, the 3D position of the radiolucent tibial polyethylene insert could be determined based on the estimated position of the tibial component. The anteroposterior translation of the points on the femoral component that were nearest to the tibial polyethylene insert (and vice versa) on the medial and lateral sides was also evaluated. External and internal axial femoral rotations were defined as positive and negative, respectively. The points on the medial and lateral sides of the femoral component that were nearest to the tibial polyethylene insert (as the center of quasi-contact) were determined by calculating the distances between the surfaces of the femoral and tibial components using CAD models. Regarding the anteroposterior positioning of the femoral component, positions anterior to the tibial insert were denoted as positive, and positions posterior to the tibial insert were regarded as negative. Valgus/varus angles (varus angles were considered to be positive) were also evaluated. We defined post/cam engagement as when the distance between the post and cam was less than 1 mm. All data are expressed as mean ± SD values. Welch’s *t* test was used for comparisons of the degree of anteroposterior displacement of the medial and lateral condyles or the valgus/varus angle. Values of *P* < 0.05 were considered to be statistically significant.

## Results

The minimum flexion angle between the femoral and tibial components was 5.2° ± 5.7° (−10.7°–13.2°) during stepping up movements and 4.0° ± 7.1° (−10.9°–11.7°) during stepping down movements. In addition, the maximum flexion angle was 45.3° ± 8.3° (22.7°–56.9°) during stepping up movements and 45.5° ± 5.6° (34.5°–55.8°) during stepping down movements.

**Fig. 2:**
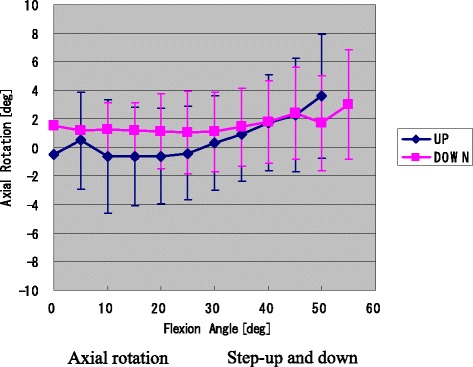
Axial rotation (mean ± SD) during stepping up and down

The axial rotation angle of the femoral component relative to the tibial component was 4.0° ± 1.4° (1.8°–6.4°) during stepping up movements and 3.2° ± 2.1° (0.6°–8.2°) during stepping down movements. The mean axial rotation of the femoral component exhibited gradual external rotation during the transition from 30° knee flexion to maximum flexion when the patients were performing stepping up movements (Fig. 2).

**Fig. 3:**
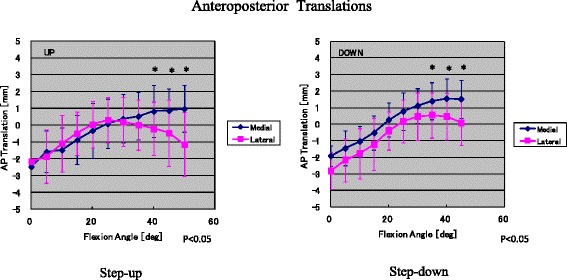
Anteroposterior translations (mean ± SD) of the medial and lateral condyle nearest points during stepping up and down

During the transition from minimum flexion to maximum flexion, medial anteroposterior translation of 2.7 ± 1.2 mm (1.0–6.2 mm) and 3.2 ± 0.9 mm (1.8–5.6 mm) was seen during stepping up and stepping down movements, respectively. In addition, lateral anteroposterior translation of 2.7 ± 1.4 mm (0.7–5.9 mm) and 3.1 ± 1.3 mm (1.5–6.1 mm) was observed during stepping up and stepping down movements, respectively. In the range from 30° knee flexion to maximum flexion, the lateral condyle exhibited slightly greater posterior rollback than the medial condyle during stepping up movements. No posterior rollback of the medial or lateral condyle occurred during stepping down movements. Slight anterior femoral translation was noted during the transition from minimum to maximum flexion (Fig. 3).

The kinematic patterns of the patient’s prostheses were determined based on the positions of the medial and lateral condyles at each flexion angle. During the transition from 30° flexion to maximum flexion, a medial pivot-type kinematic pattern involving very little anteroposterior translation was observed during stepping up movements. During stepping down movements, neither a pivot-shift-type nor a rollback-type kinematic pattern was seen. Slight anterior femoral translation occurred during the transition from minimum to maximum flexion.

**Fig. 4:**
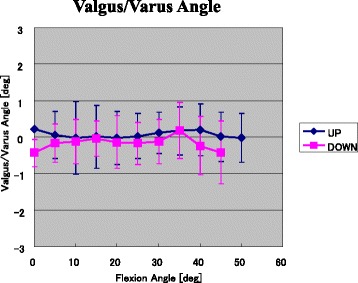
Valgus/varus angles (mean ± SD) during stepping up and down

The total valgus/varus angles for each knee were 0.1° ± 0.6° (−1.7°–1.4°) during stepping up movements and 0° ± 1.0° (−1.6°–2.6°) during stepping down movements. No significant differences in the valgus angle were detected between the two motions (Fig. 4).

Post/cam engagement was considered to have occurred in one case during stepping up movements. The minimum flexion angle seen during stepping up movements was 55.1°.

## Discussion

TKA has been demonstrated to achieve successful clinical outcomes in patients with osteoarthritis of the knee. Knee implants partially replicate the intrinsic constraints of the lost joint. However, they do not necessarily restore normal joint stability and motion so it is necessary to understand in vivo knee motion during daily activities in patients with knee prostheses.

In normal knees, the femur exhibits a medial pivot motion relative to the tibia during deep knee flexion [8, 9]. However, such movements are not always seen after TKA [10–14]. For example, Dennis reported that both medial pivot-type and lateral pivot-type patterns were seen in patients that had undergone TKA [14]. Banks found that in patients that undergo successful TKA, knee motion is directly related to the constraints of the implant [15]. On the other hand, while the center of rotation is predominantly on the lateral side of the knee during walking, the normal function of the knee during walking is associated with lateral and medial pivoting [16].

Among daily activities, the ability to use stairs is very important, as is gait. It is more important that knee prostheses exhibit good performance and stability during stair stepping than during walking. Banks reported that most patients that underwent PS TKA exhibited medial central rotation, which was indicative of posterior femoral translation and flexion, during stair stepping [17]. In our study, the subjects displayed a medial pivot kinematic pattern involving very little anteroposterior translation during stepping up movements. During stepping down movements, neither a pivot-shift-type nor a rollback-type kinematic pattern was seen. Only slight anterior femoral translation occurred during the transition from minimum to maximum flexion. The motion pattern may be caused by the tibial plate which is concave on the medial side and flat on the lateral side. The Bi-Surface PS demonstrated good joint stability during the stepping exercise. Thus, there are clear discrepancies between the kinematic patterns detected in our study and those described in Banks’ report. There were some differences in the step height and stepping method between our study and that conducted by Banks; however, we consider that the main reason for the abovementioned differences in the kinematic patterns is the unique design of the Bi-Surface PS; i.e., it is a posterior-cruciate ligament-substituting prosthesis with a characteristic ball-and-socket joint that links its femoral and tibial components.

The post/cam mechanism of the Bi-Surface PS-type implant is designed to function from 45° to 60° of knee flexion, and the ball-and-socket joint functions as the main load supporting surface from 90° of flexion. Post/cam engagement was considered to have occurred in one case during stepping up movements. The minimum flexion angle was 55.1° during stepping up movements. Furthermore, the ball-and-socket joint did not function in any case. Thus, the joint stability of the Bi-Surface PS implant during step ascension/descension is affected by the design of the femorotibial joint rather than post/cam engagement or the ball-and-socket joint. In situations involving steps that are higher than 10 cm, maximum knee flexion might increase, and greater post/cam engagement and ball-and-socket joint loading might occur during knee flexion. We consider that these kinematic patterns could affect the long-term outcomes of TKA procedures involving the Bi-Surface PS. Therefore, the relationship between these kinematic patterns and clinical outcomes should be assessed in further studies involving long-term follow-up.

## Conclusions

In summary, in patients that had undergone TKA procedures involving the Bi-Surface PS, a medial pivot kinematic pattern involving very little anteroposterior translation was seen during stepping up movements. During stepping down movements, neither a pivot-shift-type nor a rollback-type kinematic pattern was seen. Slight anterior femoral translation occurred during the transition from minimum to maximum flexion. The Bi-Surface PS demonstrated good joint stability during a stepping exercise. The joint’s stability was affected by the design of the femorotibial joint rather than post/cam engagement or the function of the ball-and-socket joint.
